# Phase analysis and mechanical dispersion perform equally in the detection of myocardial scar on cine magnetic resonance imaging

**DOI:** 10.1186/1532-429X-13-S1-P123

**Published:** 2011-02-02

**Authors:** Eva Maret, Monika Liehl, Lars Brudin, Tim Todt, Jan E Engvall

**Affiliations:** 1 Ryhov County Hospital, Jonkoping, Sweden; 2 Kalmar County Hospital, Kalmar, Sweden; 3 Linkoping University, Linkoping, Sweden

## Introduction

Myocardial contraction is a cyclic event. Contraction delay can be described in terms of mechanical dispersion or phase delay of velocity, displacement and local deformation (strain) of segments in apical views of the left ventricle. Our hypothesis was that an increase in the standard deviation of phase would be seen as a consequence of myocardial scar and that this effect could be comparable to mechanical dispersion.

## Purpose

The aim of the study was to perform phase analysis of wall motion from cine magnetic resonance (MR) images to detect myocardial scar defined with gadolinium enhanced MR.

## Methods

Thirty patients (3 women and 27 men) were selected based on the presence or absence of extensive scar in the antero- and inferoseptal areas of the left ventricle. The patients were investigated in stable clinical condition, 4-8 weeks post ST-elevation myocardial infarction treated with percutaneous coronary intervention. Seventeen had a scar area >75% in at least one anteroseptal segment (scar) and thirteen had scar <1% (non-scar). Velocity, displacement and strain as well as their respective phase delays were measured in the longitudinal direction, tangential to the endocardial outline, and in the radial direction, perpendicular to the tangent.

## Results

**Figure 1:**
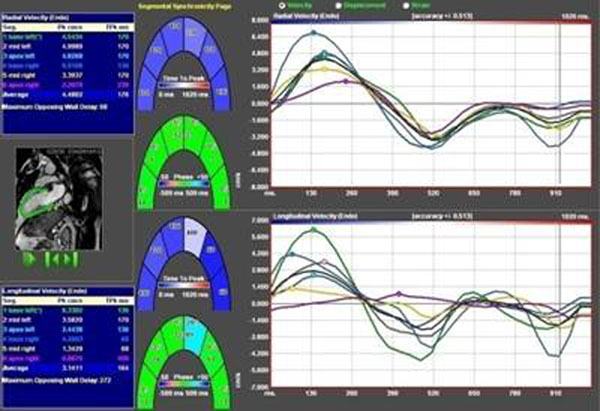
Myocardial peak velocity and time-to-peak for radial (upper) and longitudinal (lower) direction in the boxes to the left. Color coded segmental presentation of phase and time-to-peak in the middle. Line diagram of temporal changes of velocity in the radial (upper) and longitudinal (lower) direction per segment to the right.

In the scar patients, the root sum square of the standard deviations of phase in all radial measurements clearly differentiated scar patients from those without scar (p<0.001), while longitudinal measurements did so only for strain. Likewise, the standard deviation for radial measurements of time to peak (mechanical dispersion) for segmental velocity, displacement and strain all differentiated scar patients from non-scar (p<0.001), while longitudinal measurements only identified scar patients on the strain curves. Figure 1

## Conclusions

Phase delay in deformation imaging cine-MRI may be used for detecting the presence of myocardial scar in patient based analysis.

